# Supplementary figures and images for: Fast-spiking interneuron detonation drives high-fidelity inhibition in the olfactory bulb
Source: PLoS Biol. 2024 Aug 26;22(8):e3002660. doi: 10.1371/journal.pbio.3002660 (PMC11379389; doi:10.1371/journal.pbio.3002660)

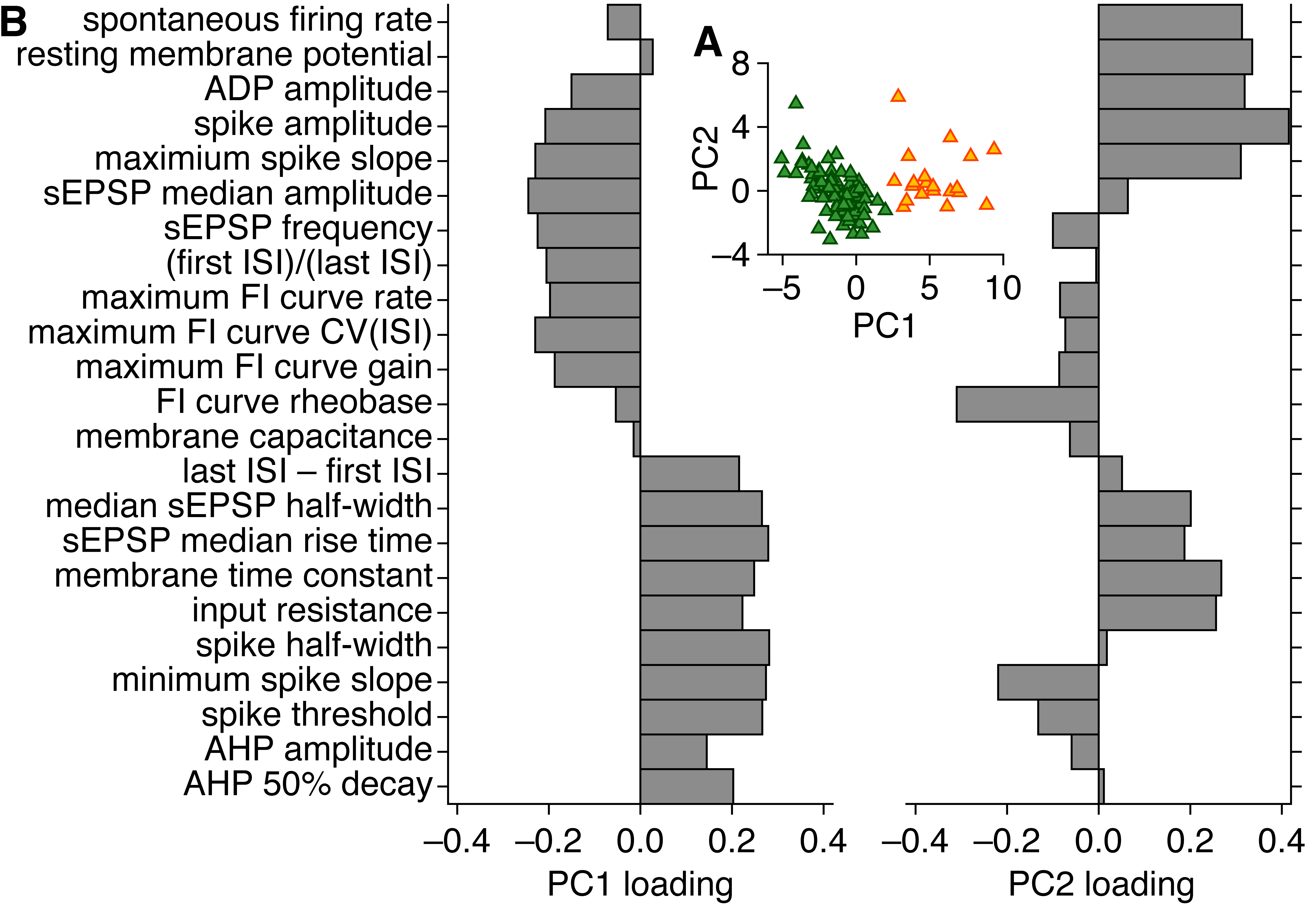

Supplement: S1 Fig — (A) Projection of 104 EPL-INs onto the first 2 principal components (PC1 and PC2) defined by principal component analysis of z-scored intrinsic biophysical properties, revealing 2 major clusters matching FSI (green) and RSI (orange) subtypes. (B) Decomposition of PC1 and PC2 loading by each intrinsic biophysical property. Source data for panels A and B are provided in Supporting information, S7 Data. (TIF) [file pbio.3002660.s001.tif]

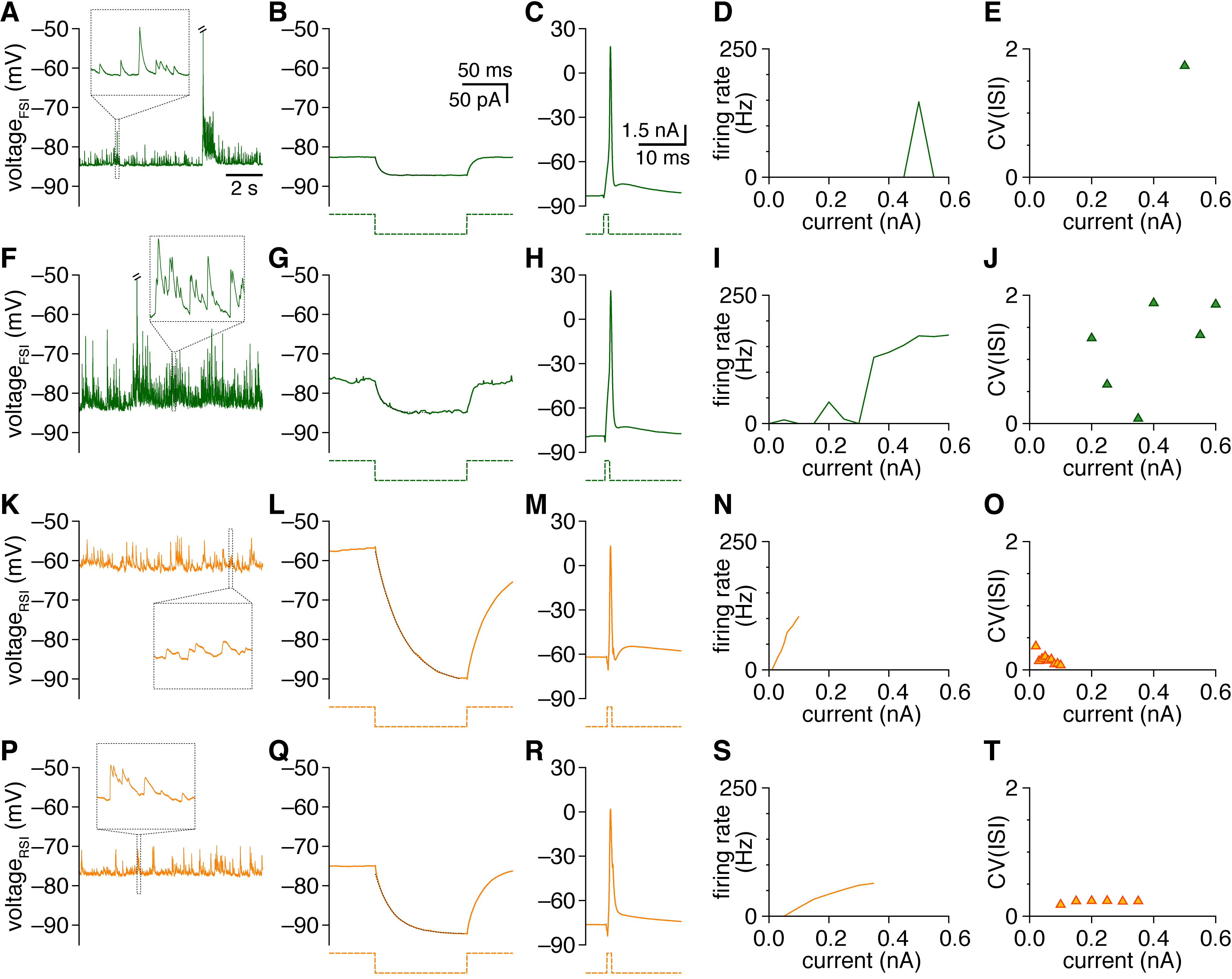

Supplement: S2 Fig — (A–E) Diverse responses used to calculate intrinsic biophysical properties for the example FSI from Fig 1B, including: spontaneous activity at resting membrane potential (A), mean response to negative step current injection, with single-exponential fit (dashed black line) (B), mean spike waveform evoked by 1-ms suprathreshold current injection (C), firing rate-current relationship (D), and interspike interval (ISI) coefficient of variation evoked by positive step current injection (E). Spontaneous spike in A truncated to better visualize synaptic activity. Inset in A: enlargement of boxed region. F–T: Same as A–E for the example FSI and RSIs from Fig 1C–1E. Insets in A, F, K, P are identically scaled. Source data for panels D, E, I, J, N, O, S, and T are provided in Supporting information, S8 Data. (TIF) [file pbio.3002660.s002.tif]

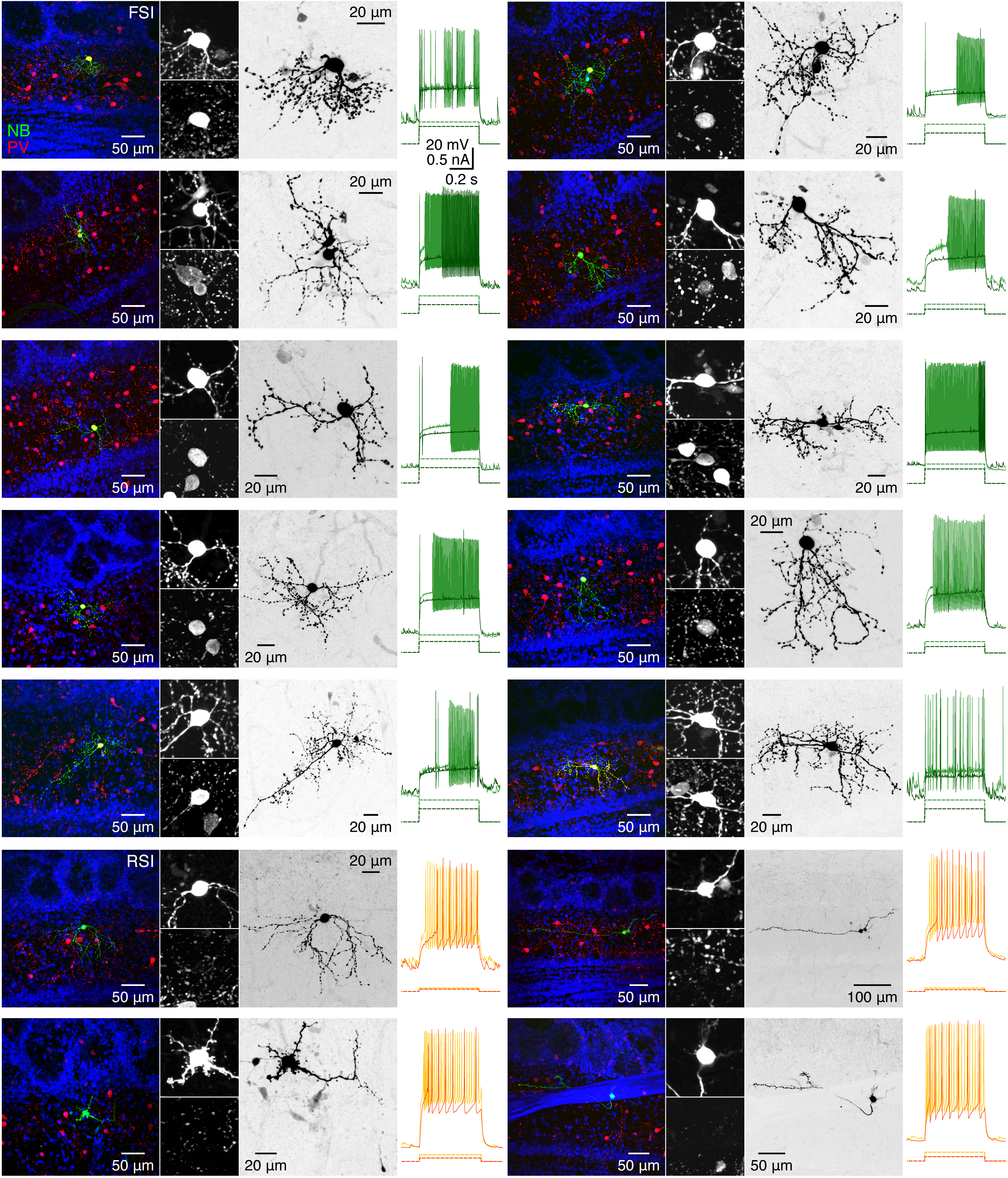

Supplement: S3 Fig — Intracellular NB and post hoc PV staining with 50-μm magnified region centered on somata (left), inverted NB (middle), and step current-evoked spiking (right) of a panel of EPL-INs. Spiking responses are color-coded to reflect FSI vs. RSI physiology, as in Fig 1. (TIF) [file pbio.3002660.s003.tif]

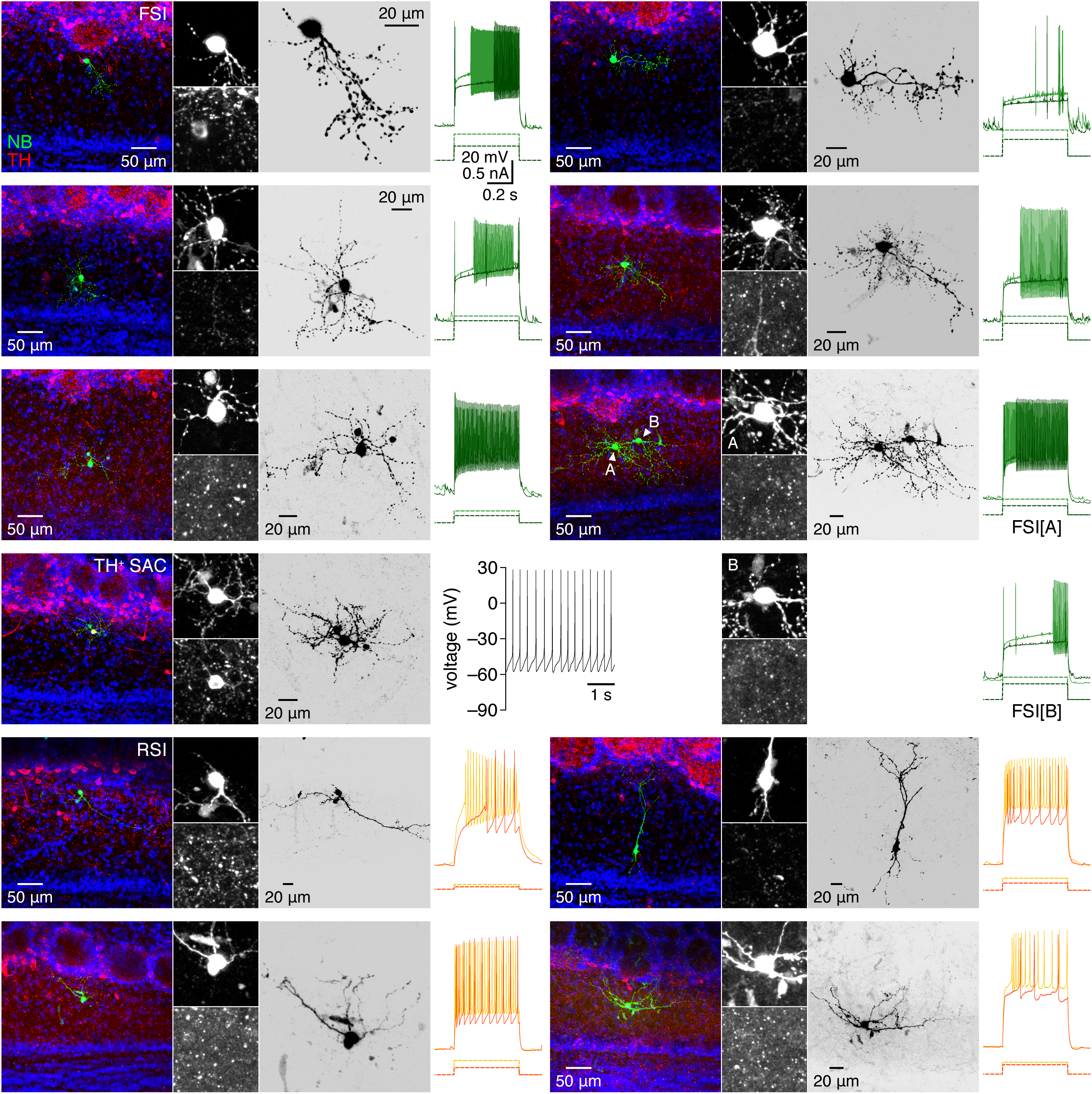

Supplement: S4 Fig — Intracellular NB and post hoc TH staining with 50-μm magnified region centered on somata (left), inverted NB (middle), and step current-evoked spiking (right) of a panel of EPL-INs. Spiking responses are color-coded to reflect FSI vs. RSI physiology, as in Fig 1. An example TH+ short-axon cell (SAC) exhibiting tonic spontaneous firing is additionally included as positive control for TH staining. (TIF) [file pbio.3002660.s004.tif]

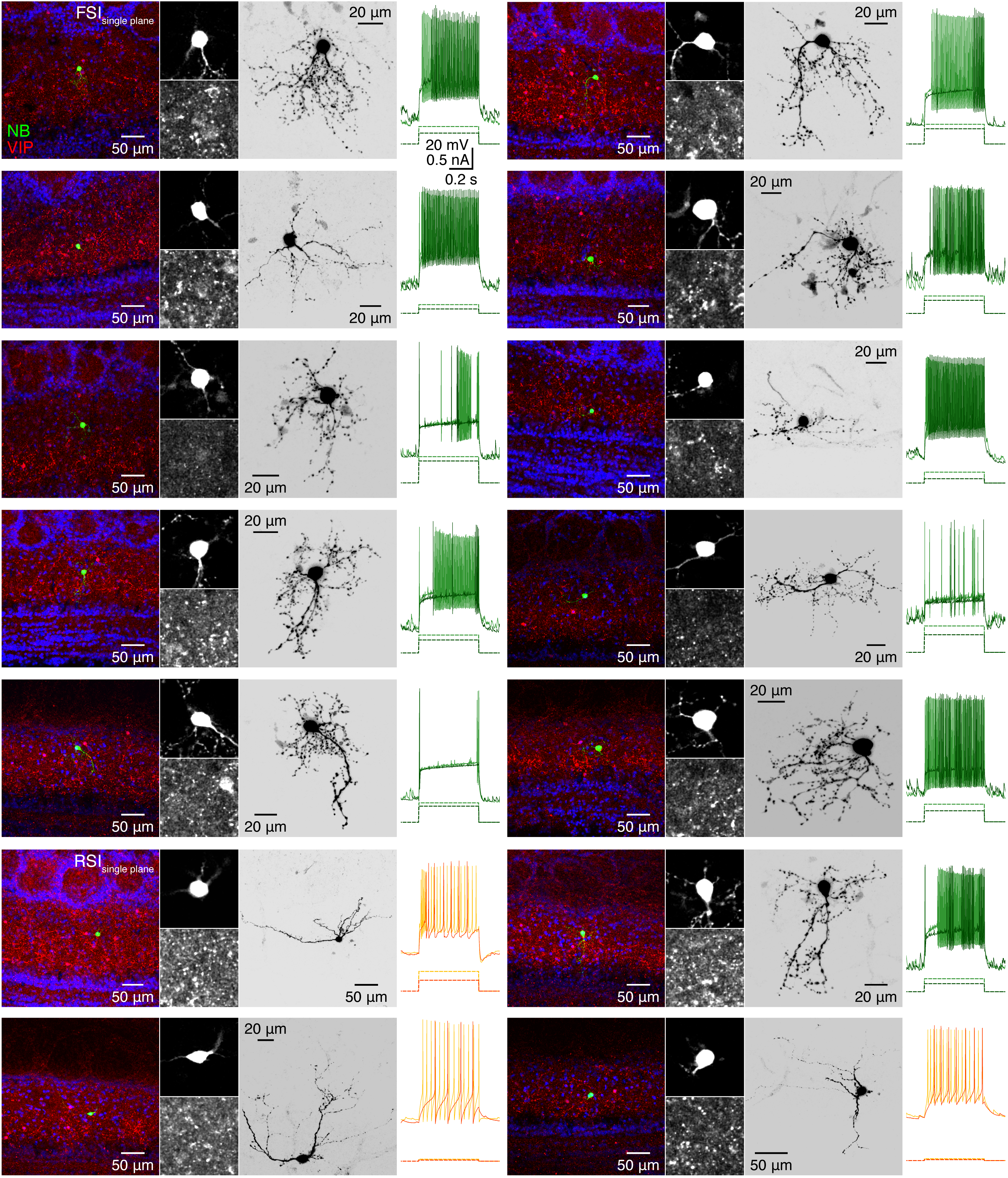

Supplement: S5 Fig — Intracellular NB and post hoc VIP staining with 50-μm magnified region centered on somata (left; single optical confocal planes), inverted NB (middle; maximum-intensity confocal projection), and step current-evoked spiking (right) of a panel of EPL-INs. Spiking responses are color-coded to reflect FSI vs. RSI physiology, as in Fig 1. (TIF) [file pbio.3002660.s005.tif]

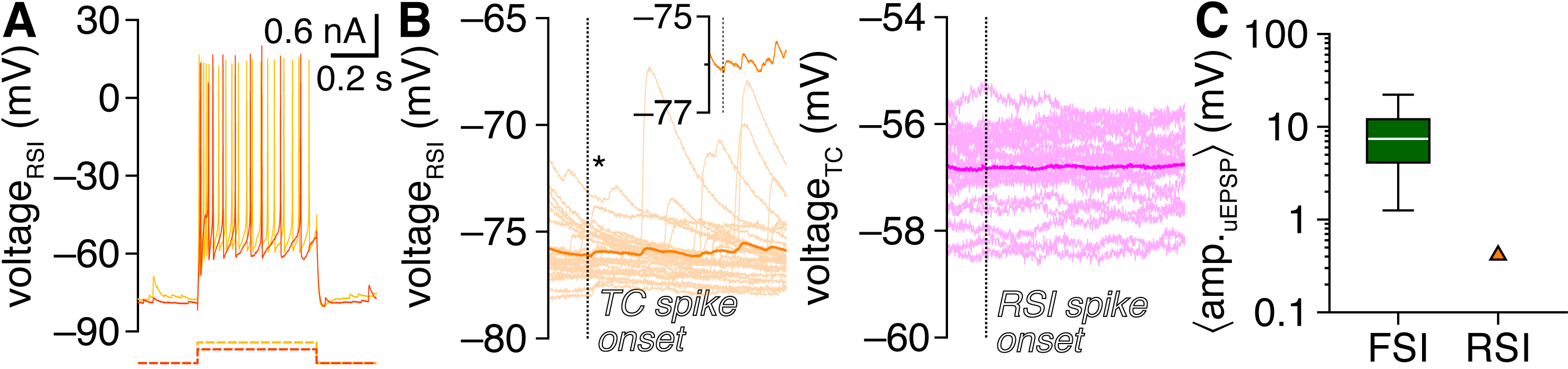

Supplement: S6 Fig — (A, B) Step current-evoked spiking response (A) and unitary synaptic interactions (B) for the solitary MTC–RSI pair exhibiting significant unitary MTC-to-RSI excitation (morphology not recovered). Asterisk marks significant postsynaptic response. Inset: mean postsynaptic RSI voltage. (C) The MTC-to-RSI uEPSP amplitude was markedly weaker than FSI uEPSPs (n = 69). Source data for panel C are provided in Supporting information, S9 Data. (TIF) [file pbio.3002660.s006.tif]

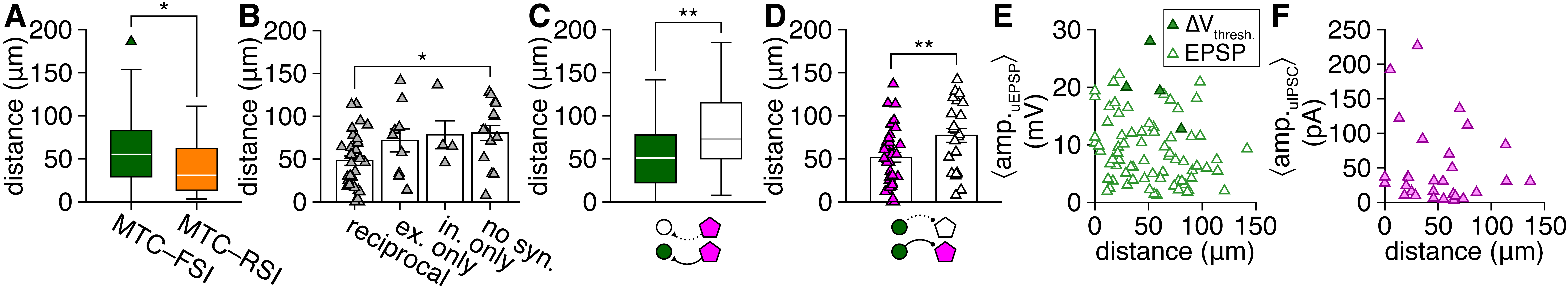

Supplement: S7 Fig — (A) MTC–RSI pairs (n = 19) exhibited modestly shorter intersomatic distances than MTC–FSI pairs (n = 97) (*p = 0.02, r.s. = 808, Wilcoxon rank-sum test). (B) Among MTC–FSI pairs, reciprocally connected pairs exhibited shorter intersomatic distances than unconnected pairs (p = 0.02, F3,55 = 3.4, one-way ANOVA; reciprocal (n = 32) vs. excitation only (n = 9): p = 0.3, reciprocal vs. inhibition only (n = 4): p = 0.4, reciprocal vs. unconnected (n = 14): p = 0.03, excitation only vs. inhibition only: p = 1.0, excitation only vs. unconnected: p = 0.9, inhibition only vs. unconnected: p = 1.0, post hoc Tukey–Kramer test). (C) MTC–FSI pairs with significant unitary MTC-to-FSI excitation (n = 71) exhibited shorter intersomatic distances than pairs with no excitatory connectivity (n = 26) (**p = 2.5 × 10^–3^, r.s. = 3,108, Wilcoxon rank-sum test). (D) MTC–FSI pairs with significant unitary FSI-to-MTC inhibition (n = 36) exhibited shorter intersomatic distances than pairs with no inhibitory connectivity (n = 23) (**p = 9.7 × 10^–3^, t57 = 2.7, two-sample t test). Analysis restricted to pairs with voltage-clamped MTCs (and therefore sensitive detection of unitary inhibition). (E, F) Neither MTC-to-FSI uEPSP amplitudes (E) nor FSI-to-MTC uIPSC amplitudes (F) correlated with intersomatic distance (uEPSP: n = 77; p = 0.1, t75 = 1.7, linear regression, slope not significantly different from 0; uIPSC: n = 29; p = 0.4, t27 = 0.8, linear regression, slope not significantly different from 0). Pairs lacking connectivity (i.e., uEPSP or uIPSC amplitude of zero) not included in analysis. Source data for all panels are provided in Supporting information, S10 Data. (TIF) [file pbio.3002660.s007.tif]

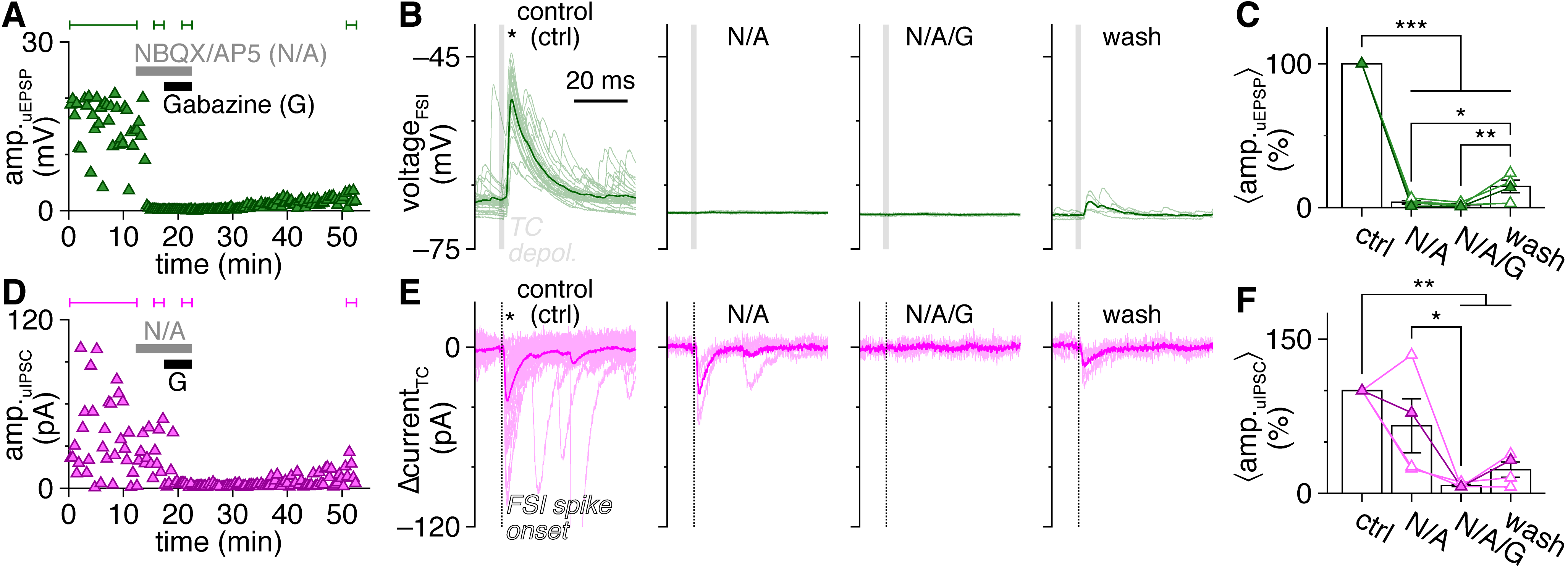

Supplement: S8 Fig — (A) Recording from an example MTC–FSI pair (morphology not recovered) showing MTC-to-FSI uEPSP amplitudes before and after combined bath application of glutamatergic antagonists NBQX (10 μM) and AP5 (50 μM) and subsequent application of GABAAR antagonist gabazine (10 μM). (B) Postsynaptic FSI voltages from the pair in A. Traces in each subplot correspond to the bracketed trials in A. Asterisk marks significant unitary postsynaptic response measured during control trials. (C) Unitary MTC-to-FSI excitation was blocked by combined application of NBQX and AP5 and partially recovered upon wash-out in 4 MTC–FSI pairs (p = 2.2 × 10^–12^, F3,12 = 414.9, one-way ANOVA; ctrl vs. N/A: ***p = 5.5 × 10^–9^, ctrl vs. N/A/G: ***p = 5.5 × 10^–9^, ctrl vs. wash: ***p = 5.5 × 10^–9^, N/A vs. N/A/G: p = 0.9, N/A vs. wash: *p = 0.03, N/A/G vs. wash: **p = 0.01, post hoc Tukey–Kramer test). (D, E) Same as A and B for FSI-to-MTC uIPSCs recorded in the same example pair. (F) Unitary FSI-to-MTC inhibition was blocked by application of gabazine and partially recovered upon wash-out in the same 4 MTC–FSI pairs as C (p = 1.7 × 10^–3^, F3,12 = 9.5, one-way ANOVA; ctrl vs. N/A: p = 0.33, ctrl vs. N/A/G: **p = 2.1 × 10^–3^, ctrl vs. wash: **p = 8.3 × 10^–3^, N/A vs. N/A/G: *p = 0.046, N/A vs. wash: p = 0.17, N/A/G vs. wash: p = 0.85, post hoc Tukey–Kramer test). Filled symbols in C and F correspond to the example pair shown. Source data for panels A, C, D, and F are provided in Supporting information, S11 Data. (TIF) [file pbio.3002660.s008.tif]

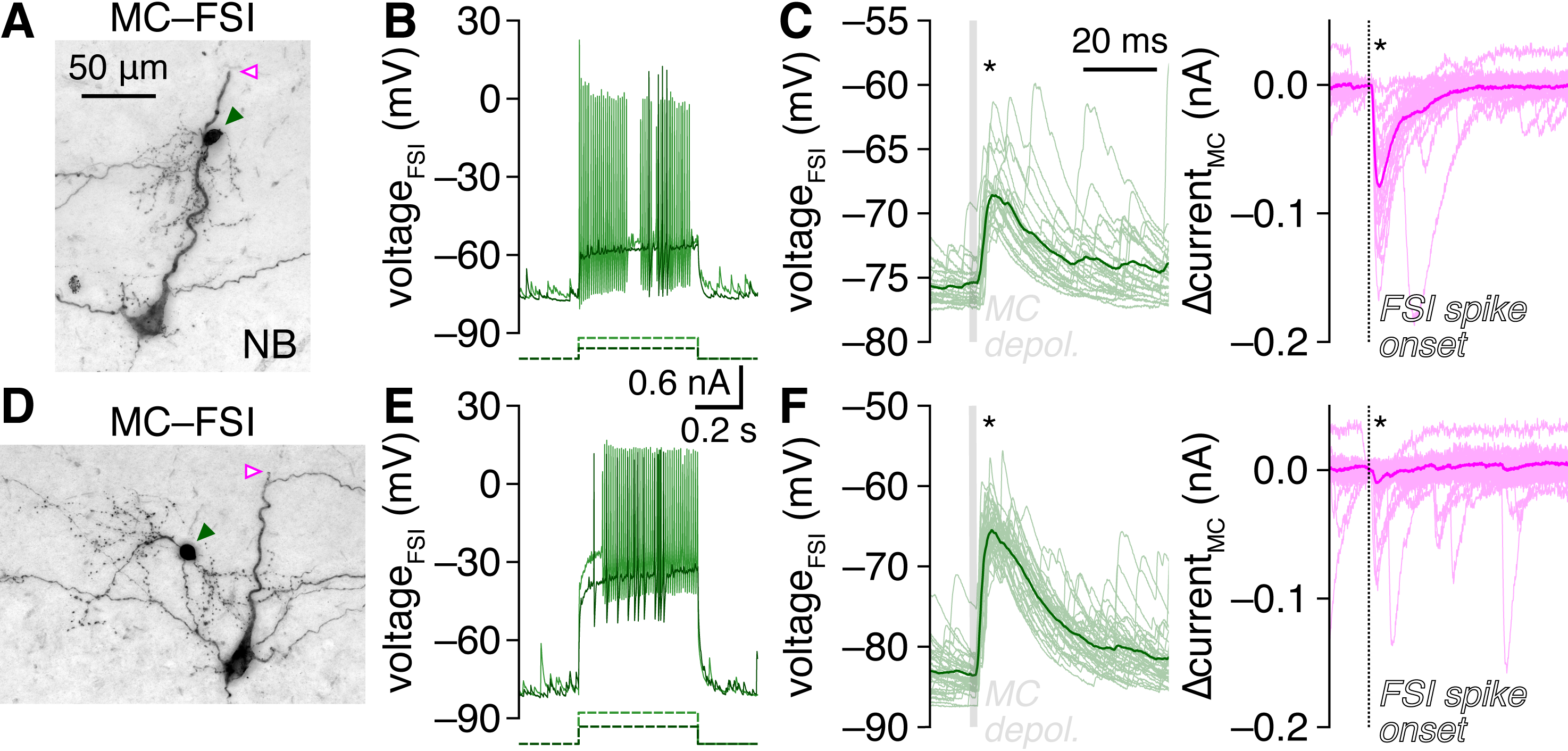

Supplement: S9 Fig — (A) Example MTC–FSI pair with MTC apical dendrite truncated prior to entering glomerular layer (open arrowhead). (B, C) FSI fast-spiking response to step current injection (B) and unitary synaptic connectivity with MTC (C). Asterisks mark significant unitary postsynaptic responses. (D–F) Same as A–C for a second example MTC–FSI pair. (TIF) [file pbio.3002660.s009.tif]

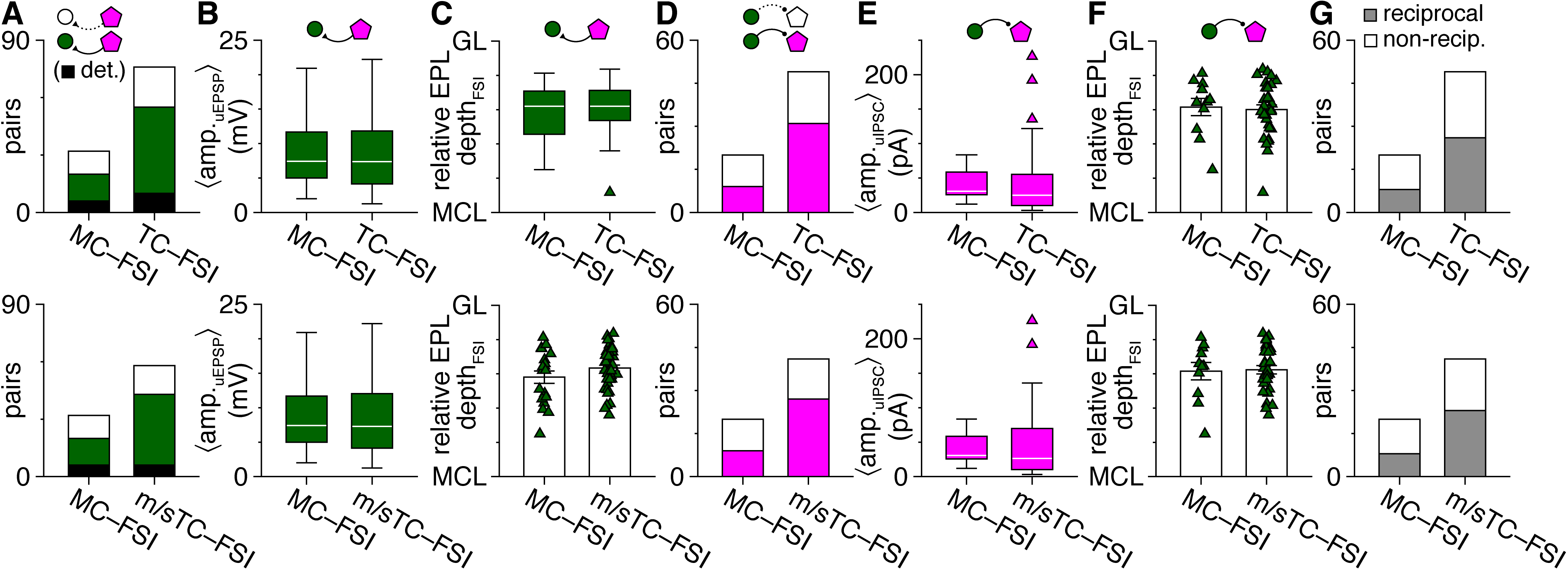

Supplement: S10 Fig — (A) Detection of unitary FSI excitation did not significantly differ between MC–FSI and TC–FSI pairs (upper; p = 0.3, χ2[1] = 1.0, χ2 test) even when considering only middle and superficial TCs (m/sTCs) to exclude potential misclassification of deep TCs (lower; p = 0.2, χ2[1] = 1.3, χ2 test). Likewise, the proportion of FSIs responding to unitary MTC release with detonation did not significantly differ between MC–FSI and TC–FSI pairs (upper; p = 0.3, χ2[1] = 1.2, χ2 test) or between MC–FSI and m/sTC–FSI pairs (lower; p = 0.1, χ2[1] = 2.3, χ2 test). (B) FSI uEPSP amplitudes did not significantly differ between MC-FSI (n = 21) and TC-FSI pairs (n = 56) (upper; p = 1.0, r.s. = 820, Wilcoxon rank-sum test) or between MC–FSI and m/sTC–FSI pairs (n = 46) (lower, p = 1.0, r.s. = 711, Wilcoxon rank-sum test). (C) Across MTC–FSI pairs with significant unitary MTC-to-FSI excitation, FSI somatic depth throughout the EPL did not differ between MC–FSI (n = 19) and TC–FSI pairs (n = 53) (upper; p = 0.5, r.s. = 646, Wilcoxon rank-sum test) or between MC–FSI and m/sTC–FSI pairs (n = 43) (lower, p = 0.1, t60 = 1.5, two-sample t test). (D) Detection of unitary MTC inhibition did not significantly differ between MC–FSI and TC–FSI pairs (upper; p = 0.2, χ2[1] = 1.9, χ2 test) or between MC–FSI and m/sTC–FSI pairs (lower; p = 0.1, χ2[1] = 2.4, χ2 test); only voltage-clamped MTCs were considered for peak detection sensitivity. (E) MTC uIPSC amplitudes did not significantly differ between MC–FSI (n = 5) and TC–FSI pairs (n = 29) (upper; p = 0.6, r.s. = 100, Wilcoxon rank-sum test) or between MC–FSI and m/sTC–FSI pairs (n = 26) (lower; p = 0.6, r.s. = 90, Wilcoxon rank-sum test). (F) Across MTC–FSI pairs with significant unitary FSI-to-MTC inhibition, FSI somatic depth throughout the EPL did not differ between MC–FSI (n = 11) and TC–FSI pairs (n = 33) (p = 0.8, t42 = 0.3, two-sample t test) or between MC–FSI and m/sTC–FSI pairs (n = 30) (lower, p = 0.9, t39 = 0.2, two-sample t test). (G) Th [file pbio.3002660.s010.tif]

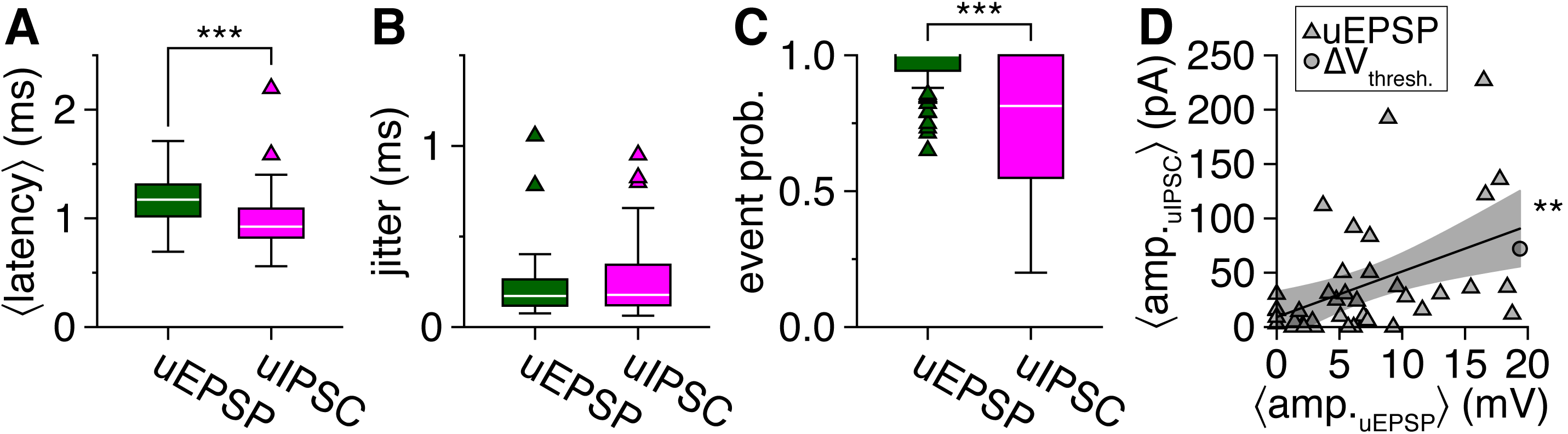

Supplement: S11 Fig — (A) FSI-to-MTC uIPSC latency (n = 44) was significantly shorter than MTC-to-FSI uEPSP latency (n = 31) (***p = 8.0 × 10^–5^, r.s. = 1,545, Wilcoxon rank-sum test). (B) FSI-to-MTC uIPSC jitter and MTC-to-FSI uEPSP jitter were equivalent (p = 0.5, r.s. = 1,117, Wilcoxon rank-sum test). (C) Trial-to-trial FSI-to-MTC uIPSC event probability (n = 44) was significantly lower than trial-to-trial MTC-to-FSI uEPSP event probability (n = 79) (***p = 5.4 × 10^–8^, r.s. = 5,838, Wilcoxon rank-sum test). Unitary FSI-to-MTC IPSP latency, jitter, and probability not included in comparisons due to limited unitary IPSP detection sensitivity (Fig 1R). (D) Across all MTC–FSI pairs with at least 1 direction of unitary connectivity, FSI-to-MTC uIPSC amplitude positively correlated with MTC-to-FSI uEPSP amplitude (n = 41; **p = 1.9 × 10^–3^, t39 = 3.3, R2 = 0.22, linear regression, slope significantly different from 0). For pairs exhibiting exclusive FSI detonation, uEPSP amplitudes were estimated as the difference between resting membrane potential and spike threshold (ΔVthresh.), as in Fig 3G. Shading denotes 95% confidence interval. Source data for all panels are provided in Supporting information, S13 Data. (TIF) [file pbio.3002660.s011.tif]

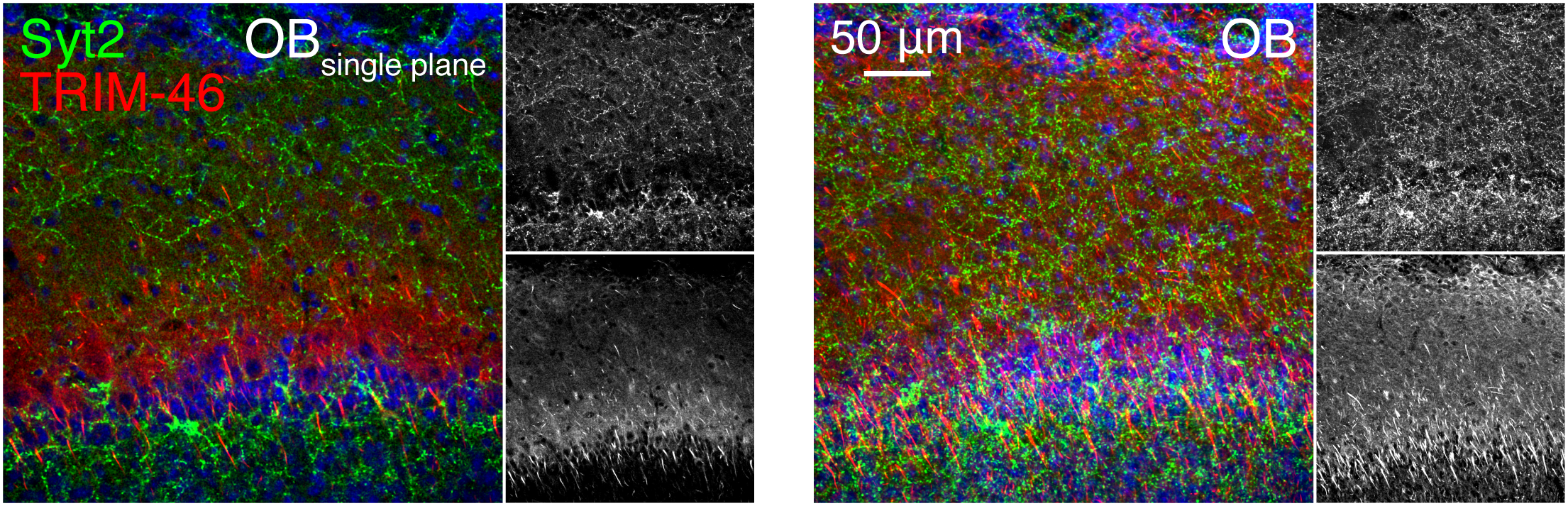

Supplement: S12 Fig — Single confocal optical plane (left) and maximum-intensity projection (right; approximately 50 μm depth) of Syt2 and axon initial segment component TRIM-46 in the OB, revealing an absence of clear Chandelier-like innervation of MTCs. (TIF) [file pbio.3002660.s012.tif]

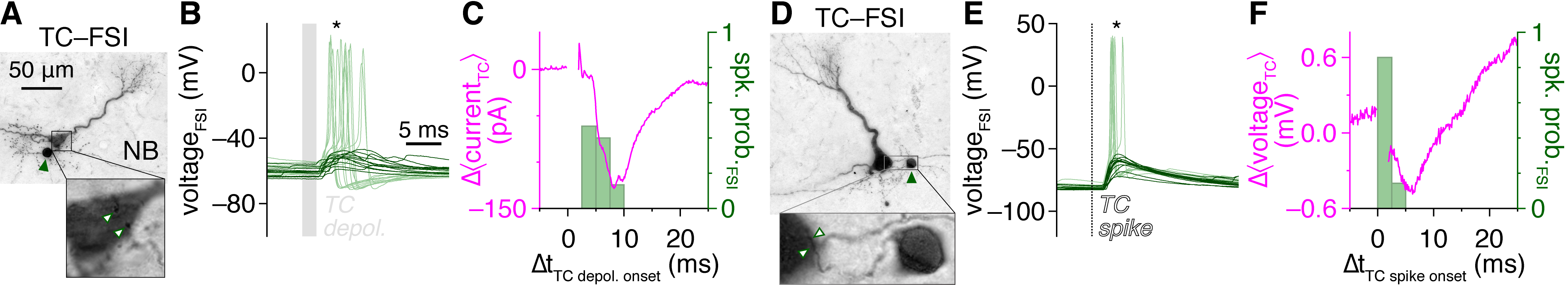

Supplement: S13 Fig — (A, B) Example MTC–FSI pair (A) in which unitary MTC release triggers FSI detonation on some trials (light green) and uEPSPs on other trials (dark green) (B). Asterisk marks significant unitary postsynaptic response. (C) Subtraction of mean MTC currents across FSI detonation vs. uEPSP trials from B isolates IPSC waveforms time-locked to FSI detonation. (D–F) Same as A–C for an example MTC-FSI pair recorded in current-clamp, revealing isolation of an IPSP waveform time-locked to FSI detonation. (TIF) [file pbio.3002660.s013.tif]

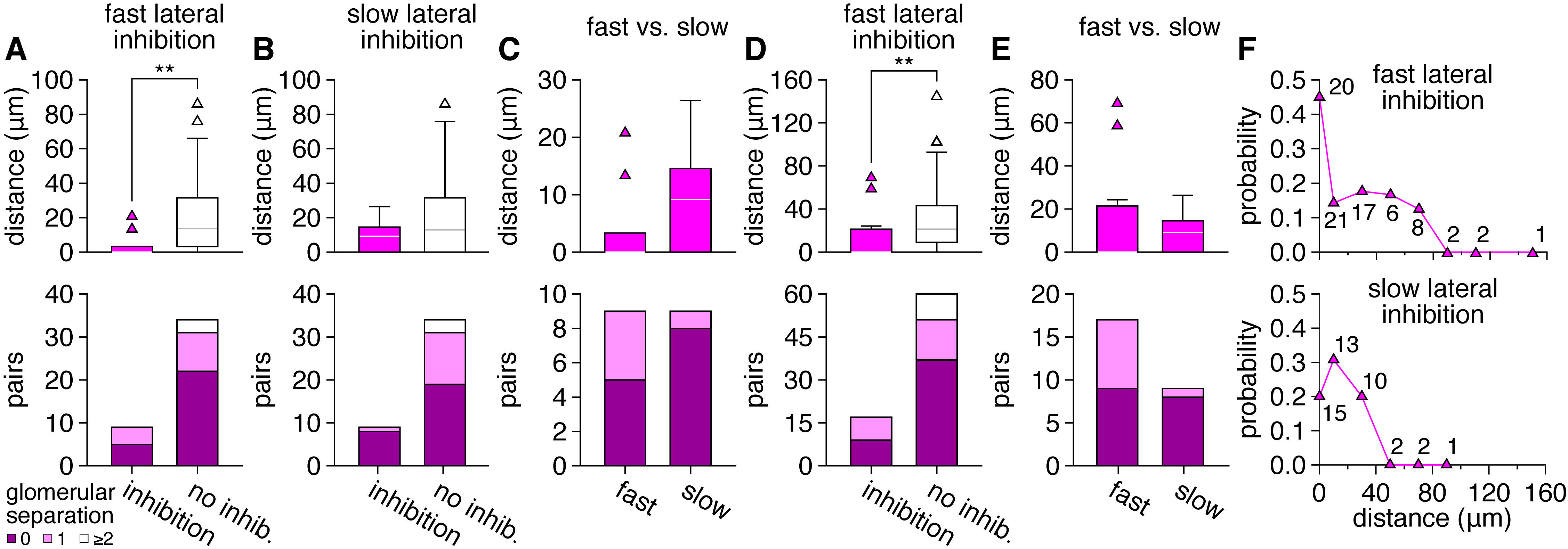

Supplement: S14 Fig — (A) Among heterotypic MTC pairs tested for both fast and slow lateral inhibition (Fig 5), pairs with fast lateral inhibition (n = 9) exhibited shorter intersomatic distances than pairs without fast lateral inhibition (n = 34) (**p = 8.3 × 10^–3^, r.s. = 111, Wilcoxon rank-sum test) (upper). No difference was observed in the number of glomeruli separating the apical dendrites of MTCs with vs. without fast lateral inhibition (p = 0.4, χ2[2] = 1.6, χ2 test) (lower). (B) Pairs with slow lateral inhibition (n = 9) exhibited comparable intersomatic distances as pairs without slow lateral inhibition (n = 34) (p = 0.3, r.s. = 166, Wilcoxon rank-sum test) (upper). No difference was observed in glomerular separation (p = 0.2, χ2[2] = 3.4, χ2 test) (lower). (C) Pairs with fast lateral inhibition (n = 9) exhibited comparable intersomatic distances as pairs with slow lateral inhibition (n = 9) (p = 0.2, r.s. = 70.5, Wilcoxon rank-sum test) (upper). No difference was observed in glomerular separation (p = 0.1, χ2[2] = 2.5, χ2 test) (lower). (D, E) Same as A and C, but including heterotypic MTC pairs tested for both fast and slow lateral inhibition (Fig 5) as well as heterotypic MTC pairs comprising each quartet tested for fast lateral inhibition (Fig 6; only single presynaptic MTC activation results included). Pairs with fast lateral inhibition (n = 17) exhibited shorter intersomatic distances than pairs without fast lateral inhibition (n = 60) (**p = 9.6 × 10^–3^, r.s. = 453.5, Wilcoxon rank-sum test) (D upper) but comparable intersomatic distances as pairs with slow lateral inhibition (n = 9) (p = 0.9, r.s. = 227.5, Wilcoxon rank-sum test) (E upper); no difference was observed in glomerular separation as a function of fast lateral inhibition (p = 0.07, χ2[2] = 5.3, χ2 test) (D lower) or fast vs. slow lateral inhibition (p = 0.07, χ2[2] = 3.6, χ2 test) (E lower). (F) Probability of detecting fast lateral inhibition (upper; pairs from Figs 5 and 6) or slow lateral inhibition (lower; [file pbio.3002660.s014.tif]
