# Supplementary material for: Fast-spiking interneuron detonation drives high-fidelity inhibition in the olfactory bulb
Source: PLoS Biol. 2024 Aug 26;22(8):e3002660. doi: 10.1371/journal.pbio.3002660 (PMC11379389; doi:10.1371/journal.pbio.3002660)
Supplement: S1 Table — Source data provided in Supporting information, S15 Data. (DOCX) [file pbio.3002660.s015.docx]

# Supporting information

S1 Table. EPL-IN intrinsic biophysical properties.

|  | **FSI** | | | **RSI** | | |  |  |  |
| --- | --- | --- | --- | --- | --- | --- | --- | --- | --- |
|  | **mean ± SEM** | **median**  **(Q_1_, Q_3_)** | **N** | **mean ± SEM** | **median**  **(Q_1_, Q_3_)** | **N** | **sig.** | **p** | **test value** |
| *Resting properties* | | | | | | | | | |
| resting potential  (mV) | –81.9±0.5 | –82.8  (–84.5, –79.7) | 95 | –78.5±1.6 | –81.5  (–84.2, –73.9) | 27 | n.s. | 0.1 | r.s. = 1907.5 |
| input resistance  (MΩ) | 146.4±5.5 | 137.8  (110.0, 178.8) | 92 | 422.1±30.9 | 373.3  (288.8, 523.9) | 27 | *** | 1.0×10^–14^ | r.s. = 2840 |
| membrane time constant (ms) | 6.4±0.2 | 6.2  (5.1, 7.2) | 92 | 17.2±1.2 | 17.1  (13.0, 19.9) | 27 | *** | 1.5×10^–13^ | r.s. = 2785 |
| membrane capacitance (pF) | 46.0±1.2 | 43.7  (37.1, 54.0) | 92 | 42.8±2.6 | 43.6  (36.3, 52.2) | 27 | n.s. | 0.4 | r.s. = 1494 |
| spontaneous firing rate (Hz) | 0.2±0.1 | 0.02  (0.0, 0.1) | 95 | 0.04±0.03 | 0.0  (0.0, 0.02) | 27 | ** | 3.1×10^–3^ | r.s. = 1206 |
| spontaneous EPSP half-width (ms) | 4.2±0.1 | 4.1 (3.8,4.5) | 94 | 9.2±0.9 | 7.7 (7.2, 9.0) | 22 | *** | 8.3×10^–13^ | r.s. = 2303.5 |
| spontaneous EPSP rate (Hz) | 58.7±1.5 | 59.0  (48.7, 68.0) | 95 | 30.7±1.9 | 30.4  (23.5, 37.5) | 27 | *** | 3.7×10^–16^ | t_120_ = 9.4 |
| spontaneous EPSP median amp. (mV) | 1.8±0.1 | 1.8 (1.4, 2.1) | 95 | 0.5±0.0 | 0.5 (0.5, 0.7) | 27 | *** | 2.7×10^–14^ | r.s. = 426 |
| spontaneous EPSP median rise (ms) | 0.6±0.0 | 0.6 (0.5,0.6) | 94 | 0.9±0.0 | 0.9 (0.8, 1.1) | 23 | *** | 3.6×10^–13^ | r.s. = 2417.5 |
| *Step current-evoked spike properties* | | | | | | | | | |
| spike amp.  (mV) | 63.6±1.5 | 63.2  (53.6, 74.0) | 92 | 52.3±2.9 | 52.3  (42.8, 61.0) | 23 | ** | 1.2×10^–3^ | t_113_ = 3.3 |
| spike width  (ms) | 0.4±0.0 | 0.4  (0.4, 0.5) | 92 | 0.7±0.0 | 0.7  (0.6, 0.8) | 23 | *** | 2.6×10^–10^ | r.s. = 2210.5 |
| spike threshold  (mV) | –50.0±0.8 | –50.1  (–55.3, –45.3) | 92 | –35.3±1.6 | –35.7  (–38.8, –30.4) | 23 | *** | 4.5×10^–13^ | t_113_ = 8.2 |
| max. spike rising slope (mV/ms) | 284.6±10.6 | 269.5  (212.3, 353.0) | 92 | 179.7±18.5 | 163.1  (114.5, 228.2) | 23 | *** | 1.5×10^–5^ | r.s. = 715 |
| max. spike falling slope (mV/ms) | –187.3±6.1 | ­–182.8  (–230.5, –143.6) | 92 | –89.8±8.4 | –84.4  (–103.9, –63.9) | 23 | *** | 6.0×10^–10^ | r.s. = 2220 |
| afterhyperpolarization amp. (mV) | 19.9±0.6 | 20.0  (16.4, 24.1) | 92 | 23.8±1.0 | 22.4  (19.9, 26.4) | 23 | ** | 1.5×10^–3^ | t_113_ = 3.3 |
| afterhyperpolarization 50% decay (ms) | 5.9±0.5 | 4.6  (3.2, 7.2) | 92 | 41.7±11.8 | 18.2  (9.9, 54.4) | 22 | *** | 2.1×10^–8^ | r.s. = 2046 |
| *Pulse-evoked spike properties* | | | | | | | | | |
| afterdepolarization amp. (mV) | 1.7±0.1 | 1.4  (0.6, 2.7) | 93 | 0.4±0.4 | 0.0  (0.0, 0.0) | 22 | *** | 1.7×10^–8^ | r.s. = 492 |
| *Firing rate-current (FI) curve properties* | | | | | | | | | |
| rheobase  (pA) | 166.1±9.7 | 150.0  (100.0, 200.0) | 93 | 98.9±12.3 | 100.0  (35.0, 150.0) | 27 | *** | 9.2×10^–4^ | r.s. = 1114 |
| max. gain  (Hz/pA) | 2.1±0.1 | 2.0  (1.4, 2.6) | 93 | 0.9±0.2 | 0.6  (0.4, 1.0) | 27 | *** | 4.9×10^–9^ | r.s. = 702 |
| max. instantaneous rate (Hz) | 243.6±5.7 | 238.1  (204.1, 277.8) | 93 | 147.9±12.1 | 149.3  (99.5, 180.2) | 27 | *** | 3.7×10^–12^ | t_118_ = 7.7 |
| max. interspike interval (ISI) C.V. | 2.2±0.1 | 2.3  (1.4, 3.1) | 92 | 0.3±0.0 | 0.3  (0.2, 0.4) | 27 | *** | 9.6×10^–14^ | r.s. = 446 |
| relative adaptation (ISI_first_/ISI_last_; %) | 94.5±1.9 | 92.4  (86.8, 101.4) | 88 | 58.4±5.2 | 49.9  (41.0, 73.7) | 27 | *** | 2.3×10^–8^ | r.s. = 719 |
| absolute adaptation (ISI_last_–ISI_first_; ms) | 0.7±0.2 | 0.7  (–0.1, 1.2) | 88 | 26.0±4.4 | 22.4  (9.6, 44.2) | 27 | *** | 1.4×10^–9^ | r.s. = 2484 |
