# Supplementary material for: Fast-spiking interneuron detonation drives high-fidelity inhibition in the olfactory bulb
Source: PLoS Biol. 2024 Aug 26;22(8):e3002660. doi: 10.1371/journal.pbio.3002660 (PMC11379389; doi:10.1371/journal.pbio.3002660)
Supplement: S2 Table — Source data provided in Supporting information, S16 Data. (DOCX) [file pbio.3002660.s016.docx]

# Supporting information

S2 Table. EPL-IN anatomical and morphometric properties.

|  | **FSI** | | | **RSI** | | |  |  |  |
| --- | --- | --- | --- | --- | --- | --- | --- | --- | --- |
|  | **mean ± SEM** | **median**  **(Q_1_, Q_3_)** | **N** | **mean ± SEM** | **median**  **(Q_1_, Q_3_)** | **N** | **sig.** | **p** | **test value** |
| relative EPL depth (0=MCL, 1=GL) | 0.59±0.02 | 0.62  (0.49, 0.71) | 104 | 0.65±0.02 | 0.63  (0.55, 0.76) | 28 | n.s. | 0.2 | r.s. = 6659 |
| soma area  (μm^2^) | 94.2±5.2 | 97.0  (77.9, 104.8) | 16 | 78.6±5.9 | 73.8  (61.6, 96.4) | 9 | n.s. | 0.07 | t_23_ = 1.9 |
| soma max. diameter  (μm) | 13.2±0.4 | 13.2  (12.4, 13.9) | 16 | 13.3±0.8 | 12.6  (11.4, 15.0) | 9 | n.s. | 0.9 | t_23_ = 0.1 |
| soma max. diameter/ min. diameter | 1.37±0.06 | 1.32  (1.16, 1.53) | 16 | 1.48±0.11 | 1.38  (1.28, 1.50) | 9 | n.s. | 0.3 | r.s. = 189 |
| dendritic fractal dimension | 1.11±0.01 | 1.12  (1.08, 1.14) | 16 | 1.08±0.01 | 1.07  (1.05, 1.09) | 9 | * | 0.01 | t_23_ = 2.8 |
| total dendritic length (mm) | 1.44±0.07 | 1.48  (1.21, 1.68) | 16 | 0.84±0.14 | 0.71  (0.53, 1.14) | 9 | *** | 3.6×10^–4^ | t_23_ = 4.2 |
| # primary dendritic branches | 4.3±0.5 | 4.0  (3.0, 5.5) | 16 | 3.9±0.6 | 4.0  (3.0, 4.0) | 9 | n.s. | 0.7 | r.s. = 214.5 |
| # terminal dendritic branches | 50.8±4.0 | 49.5  (38.5, 63.5) | 16 | 15.7±3.8 | 11.0  (9.8, 21.5) | 9 | *** | 2.1×10^–4^ | r.s. = 274 |
| # total dendritic branches | 96.4±7.7 | 95.5  (72.5, 115.5) | 16 | 27.0±7.3 | 18.0  (15.8, 35.8) | 9 | *** | 2.0×10^–4^ | r.s. = 274 |
| dendritic spine density (#/100 μm) | 3.2±0.2 | 3.2 (2.7, 3.8) | 16 | 11.6±2.2 | 13.7  (4.4, 16.0) | 9 | *** | 3.1×10^–5^ | t_23_ = 5.2 |
