# Supplementary material for: Fast-spiking interneuron detonation drives high-fidelity inhibition in the olfactory bulb
Source: PLoS Biol. 2024 Aug 26;22(8):e3002660. doi: 10.1371/journal.pbio.3002660 (PMC11379389; doi:10.1371/journal.pbio.3002660)
Supplement: S3 Table — (DOCX) [file pbio.3002660.s017.docx]

# Supporting information

S3 Table. Template parameters for postsynaptic event detection.

| **Event** | **baseline**  **(ms)** | **duration**  **(ms)** | **amplitude**  **(a.u.)** | **rise**  **(ms)** | **decay (ms)** | **min. separation**  **(ms)** | **threshold**  **(×S.D.)** |
| --- | --- | --- | --- | --- | --- | --- | --- |
| MTC IPSC | 0.5 | 5.0 | –1 | 0.8 | 4.0 | 0.5 | 2.5 |
| MTC IPSP | 1.0 | 8.0 | –1 | 1.0 | 6.0 | 0.5 | 3.0 |
| EPL-IN EPSP | 0.5 | 5.0 | 1 | 0.7 | 4.0 | 0.5 | 2.5 |
