# Supplementary material for: Fast-spiking interneuron detonation drives high-fidelity inhibition in the olfactory bulb
Source: PLoS Biol. 2024 Aug 26;22(8):e3002660. doi: 10.1371/journal.pbio.3002660 (PMC11379389; doi:10.1371/journal.pbio.3002660)
Supplement: Attachment — Submitted filename: Burton Malyshko Urban 2024 response to reviewers.pdf [file pbio.3002660.s034.pdf]

We thank the editors and reviewers for their careful consideration of our manuscript. In responding to each of the reviewer comments, we have made numerous revisions to our original manuscript, including new text, analyses, and figure panels. We believe the resulting manuscript is substantially clearer and more comprehensive than our original submission, and will be of great interest to the field and readership of *PLOS Biology*.

To facilitate the review process, below we have included each of the reviewer comments (enumerated for cross-referencing), as well as our response (indented, for visual clarity), including a description of the corresponding edits made. We additionally include a second version of our revised manuscript with significant changes from the original submission highlighted with red underlined text.

Reviewer #1: Review of PBIOLOGY-D-24-01303R1, "Fast-spiking interneuron detonation drives high-fidelity inhibition in the olfactory bulb." This study by Burton and Colleagues uses whole-cell patch-clamp physiology and some immunohistochemistry to identify the source of temporally fast and precise inhibition of olfactory bulb output neurons. The study is noted for its extremely detailed and careful electrophysiology, which appears to resolve the conflict between previously observed M/T cell synchrony and the temporally distributed nature of inhibition they receive from granule cells. The experiments are technically demanding, and the results are convincing. The attention to detail and thoroughness are commendable; however, this is also somewhat of a weakness of the manuscript. The details and figures are rather dense, and, in several places, the overall message becomes obscured. This does not dampen my enthusiasm for the study. Still, I recommend that the authors revise their text to make it more accessible for general biologists (PLOS Biology) and not only those with a deep understanding of electrophysiological methods. Overall, this is a strong study with a remarkably detailed investigation. The authors should be able to address each of the points below without the need for further experimentation.

We have introduced several edits to render our revised manuscript more accessible for a general audience. Some of the more substantial edits include:

- New text in the revised Introduction outlines the major flow of activity through the olfactory bulb (OB) circuit (lines 40-48).
- In the revised Introduction, we have integrated a new schematic of the OB circuit (S1 Fig), depicting input from the periphery, output to cortex, and major components supporting feedforward, recurrent, and lateral MTC inhibition.
- In figures with voltage and/or current traces of potential synaptic connections, we have now marked significant postsynaptic responses with an asterisk to help direct the reader to the salient information being displayed. Explanation of this formatting has been added to the respective figure captions.
- In Fig 1U, "IPSC" and "IPSP" labels have been replaced with "VC" and "IC" (corresponding to voltage and current clamp, respectively) to more directly match the terminology used in the figure caption and in labels throughout other figures, as well as to no longer require the reader to understand that inhibitory postsynaptic currents and potentials are recorded in VC and IC, respectively.
- Per comment #7, TC subtypes (i.e., dTC/mTC/sTC) have been removed and results "reported as they apply to the collective MTC population" (lines 150-151), simplifying description of results and avoiding confusion of "MTC" with "middle TCs".
- We have updated the labeling used in Fig 3D inset from "sub." and "supra." to "uEPSP" and "detonation" to more directly match the terminology used in the text, as well as to no longer require the reader to understand that detonation reflects a suprathreshold postsynaptic response.
- We have shifted some of the electrophysiological details from the main text in the Results into figure captions or the Materials and methods to help improve the flow of the main text. For example, definition of $\Delta V_{thresh.}$ as "the difference in rheobase spike threshold and median membrane potential prior to unitary excitatory input" has been moved to the Materials and methods (lines 925-926) and in the main text replaced with the more accessible definition of "the depolarization required to reach spike threshold" (lines 314-315). Likewise, in describing Fig 4I-K, where we show the sensitivity of fast lateral inhibition to philanthotoxin-7,4, we now specify in the figure caption and not the main text that MTC pairs for this were "recorded in voltage-clamp to enhance the detection of inhibition" (line 350).

- We have integrated a final schematic (S16 Fig) to help illustrate and contextualize some of our major findings.

1) A circuit schematic to accompany the introduction would be helpful and serve as a touchstone for the rest of the study. As a related note, the schematics used in Figure 1S-U are helpful and reappear throughout the study, but a legend or description of what the shapes, colors, and lines represent would be helpful.

We have integrated a basic new schematic of the OB circuit (S1 Fig) – including input from the periphery, output to cortex, and major components supporting feedforward, recurrent, and lateral MTC inhibition – to both enhance accessibility of the manuscript to more general audiences as well as to more clearly define elements in other schematics depicting recording configurations (e.g., Fig 1A) and data analysis (e.g., Fig 1S).

We have also added the following description of the schematics first appearing in Fig 1S-U to the Fig 1 caption: "Simplified schematic here and elsewhere indicates neuron types being plotted, by color (MTC: magenta; FSI: green; RSI: orange) and shape (MTC: pentagon with triangular terminal; EPL-IN: circle with circular terminal), as well as presence vs. absence of significant postsynaptic response (solid connection and coloration vs. dotted connection and no coloration)" (lines 105-108).

As noted above, we have also integrated a final schematic (S16 Fig) to help illustrate and contextualize some of our major findings.

2) In Figure 1 part P/V, why are the voltage recordings from the MT cells so much more variable than those from the FSI or RSIs? Is this meaningful in some way? Can the axes be scaled similarly to the pannels on the left?

Regarding variability in MTC membrane potentials, we have clarified in our revised Materials and methods section: "All recorded MTCs were confirmed to have intact apical dendrites and glomerular tufts (except where noted to exclude potential glomerular layer circuit interactions), as assessed through live imaging, post hoc inspection of intracellular NB or LY, and occurrence of spontaneous long-lasting depolarizations (or associated currents) reflecting glomerulus-wide activation [Carlson et al., 2000; Schoppa et al., 2001]. Frequent long-lasting depolarizations led to greater variability in baseline membrane potential in some MTC recordings (e.g., Fig 1P,R)" (lines 734-740).

Regarding axes in Fig 1P,R, we agree that equal scaling among MTC, FSI, and RSI better facilitates direct comparison of variability in baseline membrane potentials. We provide those rescaled figure panels here (with magnified views in inset):

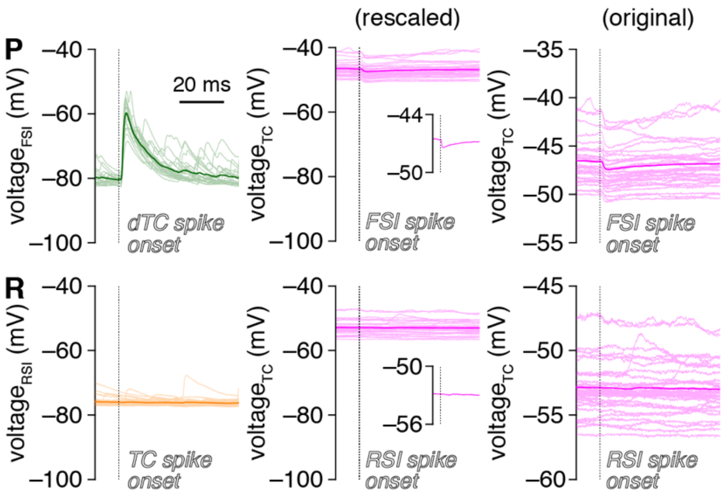

Viewed with equal scaling, the difference in membrane potential variability among MTCs, FSIs, and RSIs is fairly modest.

Reexamination of this scaling issue has re-focused our attention to the main goal of these panels: displaying the presence vs. absence of unitary postsynaptic responses. To best serve this goal, in our revised manuscript we have now individually rescaled each panel with postsynaptic responses in Fig 1 (and related S7 Fig, S10 Fig) to avoid unnecessary empty space and more clearly resolve whether a postsynaptic response occurred. In these figures, we have also marked the significant postsynaptic responses with an asterisk, as described above. Below we provide a direct comparison of original vs. revised panels from Fig 1. These revisions particularly highlight the absence of MTC–RSI connectivity, even when closely examining postsynaptic membrane potentials and currents.

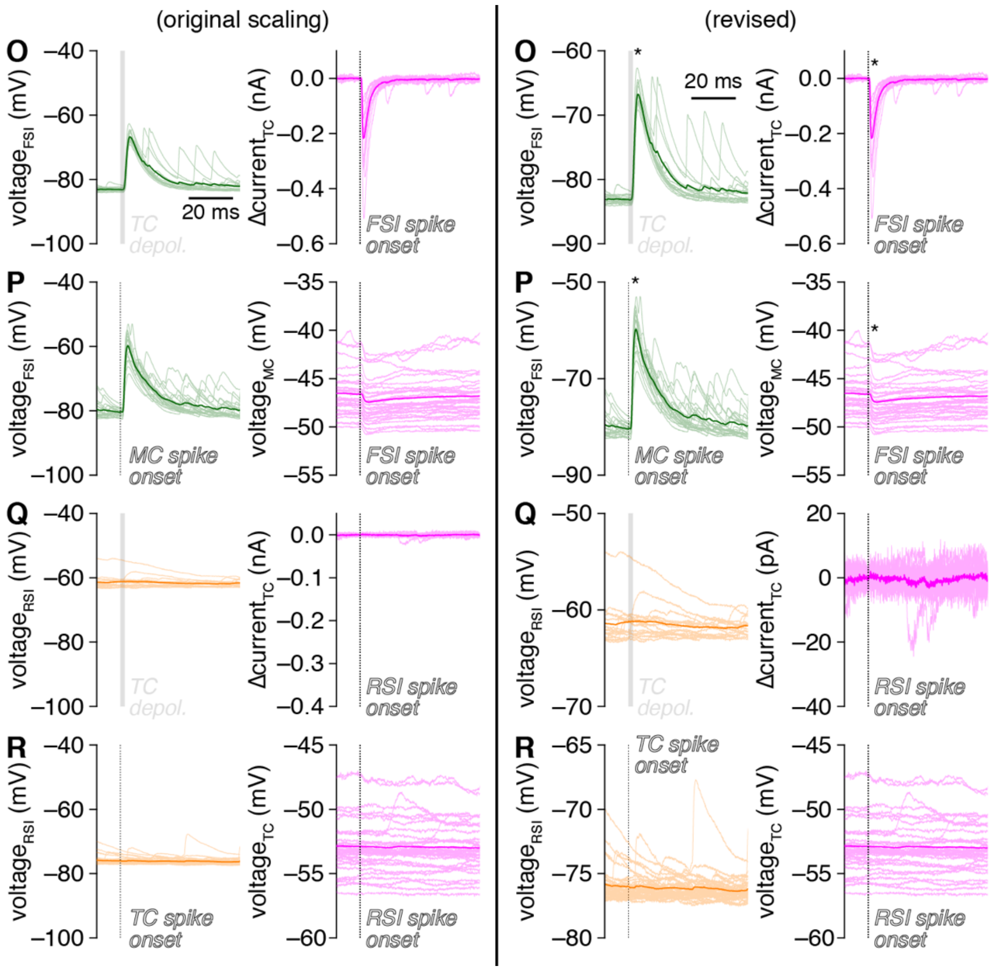

3) Line 202: the conceptual jump to cortex/hippocampus is abrupt and needs more of a transition. The authors are encouraged to explain why looking at cortical/hippocampal basket cells is analogous to looking at FSIs in the bulb. The following sentence approaches to explain the connection but is insufficient to establish the concept. "To investigate whether the evidence of perisomatic MTC innervation by single PV+ FSIs presented above collectively manifests in similar canonical patterns of basket-like innervation," Why would one expect to see patterns in the OB similar to the cortex/hippocampus? Is there previous evidence to suggest similar structures to the cortex/ hippocampus exist in the OB?

We have made multiple edits to the revised Results to improve this transition. First, we now explicitly introduce the possibility of FSI and neocortical/hippocampal basket cell similarity early in the section, immediately following initial description of MTC–FSI innervation patterns: “These patterns directly complement previous ultrastructural investigations of PV^+ EPL-INs [Toida et al., 1994, 1996; Crespo et al. 2002], suggesting that FSIs perisomatically innervate MTCs to support similar circuit operations as perisomatic-innervating interneurons elsewhere in the brain, such as PV^+ basket cells in neocortex and hippocampus” (lines 175-178). Second, we have replaced the original transition highlighted by the reviewer with a statement pointing to other FSI/basket cell similarities motivating additional comparison: “To gain further structural insight into perisomatic MTC inhibition, we next examined whether FSIs, which share several neurochemical and physiological properties with PV^+ fast-spiking basket cells throughout several regions of the brain, including neocortex and hippocampus, also innervate MTCs

with canonical patterns of profuse basket-like somatic wrapping similar to basket cell innervation of pyramidal cells” (lines 222-226).

4) Line 344: typo "enhance"

Fixed.

5) Line 281: "Strikingly, our subtraction procedure indeed isolated clear recurrent IPSCs strongly timelocked to FSI detonation latencies (Fig 3E)." Please explain in sufficient detail what the "subtraction procedure" involves.

To provide greater methodological detail, we have expanded “Strikingly, our subtraction procedure indeed isolated…” to the following: “Strikingly, subtracting the mean MTC current recorded during FSI detonation trials from the mean MTC current recorded during uEPSP trials indeed isolated…” (302-303).

We have additionally updated the labeling used in Fig 3D inset from “sub.” and “supra.” to “uEPSP” and “detonation” to more directly match the terminology used in the text.

6) While the methods section provides sufficient detail, the results section should have some more text on the optogenetic experimental design (i.e. what mice and how was stimulation achieved).

We have expanded the text in our Results section to more completely introduce this experiment, as follows: “In our final experiment, we therefore combined MTC–FSI pair recordings with activation of glomerular sensory input to assess the contribution of *single* FSIs to *total* network-driven MTC lateral inhibition. Specifically, in acute slices prepared from olfactory marker protein (OMP)-channelrhodopsin-2 (ChR2) mice, which express ChR2 in all mature olfactory sensory neurons, including their terminals within OB glomeruli [Smear et al., 2011], we recorded MTC inhibition evoked by brief photostimulation of a single nearby lateral glomerulus while, on interleaved trials, hyperpolarizing a synaptically-coupled FSI to block its feedforward excitation-evoked spiking (Fig 7A,B).” (lines 483-490)

7) The legend of Figure 7 does not patch part A. The legend states MTC-FSI pair, while the panel A label is "sTC-FSI." This type of nomenclature mismatch comes up in several of the figures. While I understand the authors are using precise descriptors of TC cell types (dTC, mTC, sTC), the distinctions are confusing when the results are generalized to MTCs. Unless there are differences found between the TC subtypes with respect to fast inhibition, I recommend simplifying the nomenclature.

As all results were found to generalize across MCs and all projection TC subtypes (S11 Fig), we have simplified our nomenclature throughout the revised manuscript, as recommended. Specifically, TC subtypes (i.e., dTC/mTC/sTC) have been removed (with the exception of S11 Fig), simplifying description of results and avoiding confusion of “MTC” with “middle TCs”. Moreover, we now explicitly state that “results are thus reported as they apply to the collective MTC population” (lines 150-151).

Overall, this is an excellent study and will be of interest to a wide range of systems neurobiologists.

Reviewer #2: This work systemically characterized the interneurons particularly the fast spiking (FSIs) type expressing parvalbumin (PV) in the external plexiform layer (EPL) of the olfactory bulb and their functional impact on output neurons with anatomical and physiological approaches. The main conclusion is that the EPL-FSIs powerfully regulate OB output via their perisomatic synapses with OB output neurons MTCs. Although morphology and physiology of the PV-expressing OB EPL interneurons were reported by a several studies, this study revealed new features of these inhibitory INs including the noncanonical perisomatic detonation, advanced our understanding their functional roles in mediating fast lateral inhibition, is especially of interest to the field of olfactory neuroscience. The manuscript is well organized with most conclusions appropriately drawn based on evidence from well-designed experiments. The Methodology has details sufficient to allow experimental reproducibility. The reviewer has only a couple concerns as listed below.

1) When the authors describe or discuss inhibition in the olfactory bulb, only GCs and EPL INs were considered, totally ignoring the inhibitory glomerular neurons. Anatomical evidence shows comparable or higher number of INs in the glomerular layer than that of GCs (Parrish-Aungst et al, 2007). The functional impact of glomerular INs on OB output was also consistently reported, especially in the past decade or so. The glomerular short axon cells are also supposed to contribute to lateral inhibition but was not mentioned in this manuscript. This treatment of the literature seems to be unfair and needs correction.

We agree that more comprehensive description of inhibitory circuits in the OB, as well as clearer qualification of our study's primary focus on EPL circuits, is warranted and would better contextualize our findings. We have thus made the following edits:

In the revised Introduction, we now provide a more comprehensive account of circuit processing in the OB, including critical operations performed by glomerular layer interneurons. We also more specifically state that conceptual models of inhibition *in the EPL* focus on GCs: "Central olfactory processing begins with transmission of sensory information from peripheral sensory neuron terminals to MTCs within odorant receptor-specific glomeruli in the OB (S1 Fig). Diverse juxtaglomerular interneurons, including large populations of inhibitory periglomerular cells [Parrish-Aungst et al., 2007], modulate this transmission to collectively support sniff-frequency coupling, gain control, and intraglomerular contrast enhancement of MTC odor responses [Wachowiak and Shipley, 2006; Cleland, 2014; Burton, 2017]. Other circuit operations critical to olfaction, such as fast timescale spike-time patterning and synchronization of MTC activity, emerge deeper in the OB, within the external plexiform layer (EPL) (Kay, 2014), where conceptual models focus exclusively on the contribution of inhibitory granule cells (GCs) to recurrent and lateral MTC inhibition" (lines 40-48).

In the revised Results, when comparing unitary FSI-to-MTC inhibitory event rise times to spontaneous event rise times, we now include juxtaglomerular interneurons in addition to GCs as sources of distal inhibition: "… consistent with FSIs innervating MTCs at more electrotonically-proximal domains than inhibition from other interneurons [Rall, 1967], such as GCs and juxtaglomerular interneurons" (lines 211-212). When introducing our experiment to assess single FSI contributions to recurrent MTC inhibition, we now more comprehensively state: "… each MTC can interact via reciprocal dendrodendritic synapses with up to 10^4 GCs [Egger and Urban, 2006; Aghvami et al., 2022] as well as a large population of periglomerular cells [Parrish-Aungst et al., 2007]" (lines 296-298). Additionally, we more specifically state that results from MTC pair and quartet recordings

only include heterotypic MTCs to "exclude the potential contribution of intraglomerular forms of lateral inhibition to the recorded currents [Najac et al., 2015]" (lines 445-446), while also noting that "we detected fast lateral inhibition in both TC pairs and MC pairs, arguing against possible contributions to fast lateral inhibition by superficial and deep short-axon cells, which selectively target TCs and not MCs [Whitesell et al., 2013; Banerjee et al., 2015; Burton et al., 2017; Vaaga et al., 2017]" (lines 367-370).

In the revised Discussion, we have amended our statement "Challenging the consensus that MTC inhibition is slow, low-fidelity, and primarily shaped by distributed changes in activity across a large population of lateral dendrite-innervating GCs" to instead include "large populations of GCs and juxtaglomerular interneurons innervating distal MTC domains" (lines 512-514). We have also removed our statement that recurrent and lateral MTC inhibition are "motifs widely attributed solely to GCs." In describing the contribution of single FSIs to MTC inhibition, we have also expanded our description of other possible sources of MTC inhibition: "… inhibition onto any MTC should conceivably also reflect the summed input from 10^4 GCs [Egger and Urban, 2006; Aghvami et al., 2022] as well as a complex array of glomerular layer circuits [Wachowiak and Shipley, 2006; Kosaka and Kosaka, 2016; Burton et al., 2017]" (lines 643-644).

2) While the authors tried to emphasize perisomatic inhibition involving somata, proximal apical dendrites and axon hillocks, synaptic interactions between EPL FSIs and lateral dendrites of MTCs cannot be completely excluded, especially for lateral inhibition. Thus, this may also need to be discussed in the text.

To clarify that our findings point toward prominent, but not exclusive, perisomatic innervation of MTCs by FSIs, we include extensive new text in our revised Discussion reviewing evidence for long-range connectivity of FSIs with distal MTC lateral dendrites, as well as how that may compare with MTC–GC connectivity:

"While our results suggest that FSIs may preferentially inhibit perisomatic MTC domains, they do not exclude FSI interactions with lateral dendrites of distal MTCs. Indeed, monosynaptic rabies viral tracing has revealed broad connectivity of PV^+ EPL-INs with MTCs, well exceeding MTC–GC connectivity ranges and consistent with broader odor-tuning of PV^+ EPL-INs than GCs [Miyamichi et al., 2013; Kato et al., 2013]. Interpretation of these results has further led to the hypothesis that proximal MTC inhibition may be primarily mediated by GCs and not by PV^+ EPL-INs [Miyamichi et al., 2013; Bartel et al., 2015; Lage-Rupprecht et al., 2020], seemingly in contrast to our present findings. As a caveat, however, the efficiency, directionality, and even specificity with which rabies transmits across dendrodendritic and dendrosomatic synapses in the OB (and how that relates to synaptic strength) remains partially unclear. For example, rabies transmits between GCs and PV^+ EPL-INs [Arenkiel et al., 2011; Miyamichi et al., 2013; Garcia et al., 2014] despite no synaptic connections [Crespo et al., 2001]. Moreover, and of particular note, while viral tracing uncovered broad MTC–PV^+ EPL-IN connectivity, the greatest density of connections was still observed among nearby MTCs and PV^+ EPL-INs [Miyamichi et al., 2013], where we have shown that FSIs perisomatically inhibit MTCs. Further, despite GC densities overall far exceeding PV^+ EPL-IN densities [Burton, 2017], the number of virally-traced short-range MTC–PV^+ EPL-IN connections well exceeded the number of short-range MTC–GC connections [Miyamichi et al., 2013], further suggesting that proximal MTC inhibition may in fact be primarily mediated by PV^+ EPL-

INs and not GCs. Our present results, revealing prominent perisomatic inhibition of MTCs by nearby FSIs, align well with this latter model. Understanding how such short-range connectivity complements long-range MTC–FSI connectivity stands as an important direction of future research (S16 Fig). Given that only ~50% of PV^+ EPL-IN synapses are reciprocal [Toida et al., 1994; 1996], key questions going forward include: 1) how common is distal MTC-to-FSI excitation, 2) how does such distal excitation compare in strength to proximal excitation, and 3) is such distal excitation matched by reciprocal FSI-to-MTC lateral dendrite inhibition? Our simultaneous whole-cell recording approach, combined with precise post hoc morphological reconstruction, is ideally suited to answer these questions." (lines 576-599)

We further point the reader to this new text following initial description of our analysis of distance-dependence: "While additional pair recordings will ultimately be required to assess the distance-dependence of MTC–FSI connectivity across intersomatic distances encompassing the full ~1 mm extent of MTC lateral dendrites (see Discussion)," (lines 202-205). Finally, in summarizing our analysis of distance-dependence in the revised Results, we now explicitly include: "… suggest that FSIs preferentially (though not exclusively) mediate perisomatic MTC inhibition" (lines 219-220).

3) Minor: cited references need to be verified. At least the following three look incomplete:
Line 1208: Marín O (2012) Interneuron dysfunction in psychiatric disorders. 13:107-120.
Line 1209-1210: Markram H, Toledo-Rodriguez M, Wang Y, Gupta A, Silberberg G, Wu C (2004) Interneurons of the neocortical inhibitory system. 5:793-807.
Line 1271: Silver RA (2010) Neuronal arithmetic. 11:474-489.

All references have now been carefully reviewed for completeness.

Reviewer #3: Effective decision making necessitates efficient processing of sensory information at behaviorally relevant time scales. Numerous studies in the rodent olfactory system over the years have conclusively shown the time scale for such processing to be on the order of 100-200 ms. In this period, recurrent circuit computations with the olfactory bulb (OB) are thought to play an important role. However, paradoxically, most of the electrophysiological studies to date have provided evidence for recurrent inhibition to be very delayed, very long lasting and low fidelity. This current work provides evidence for a form of inhibition (short latency, low jitter and high probability) that could play an essential role in bridging the gap between these two phenomena. The discovery and characterization of a population of interneurons that mediates rapid, reliable and significant lateral (and auto) inhibition of principal cells in the OB is highly significant. The experiments are extensive and extremely well done. The discovery is likely to be foundational for olfactory neuroscience. There is very little to quibble. Below are some specific comments that might be helpful.

1) Introduction, Line 40: To be fair here, the authors may want to acknowledge all the hard work on glomerular layer interneurons by people like Shipley.

> As detailed above in our response to Reviewer #2, Comment #1, we have made several edits to our revised manuscript to provide a more comprehensive description of inhibitory circuits in the OB overall, including glomerular layer interneurons and some of the many key contributions by Shipley and others.

2) Line 76: The phrasing feels clunky - just say "wild-type mice" or such?

> As recommended, we now more directly state: "… we targeted MTCs and nearby small somata of putative EPL-INs for whole-cell pair recordings in acute OB slices prepared from wild-type mice (see Materials and methods)" (lines 81-83). In the Materials and methods section, we then further clarify that "… electrophysiological experiments used multiple strains of mice on the C57BL/6J background and lacking genetic labeling of interneuron populations, and are thus considered wild-type" (lines 699-701).

3) Line 79: While the general idea of 2 big classes seems reasonable, in reality, I suspect that the number of classes is a bit dependent on hyperparameters used in any classification algorithm.

> This is an important point to convey. Indeed, our clustered heatmap of intrinsic biophysical properties (Fig 1I) suggests further heterogeneity particularly within FSIs. In addition to our Discussion section exploring EPL-IN diversity and the potential existence of further subtypes (lines 531-540 in particular), in our revised manuscript we now include this caveat quickly following description of the hierarchical clustering results:
>
> "As a caveat, such hierarchical clustering approaches can be sensitive to the specific algorithms and parameter spaces used." (lines 113-114).

4) Line 88 and other places further down: Instead of "Magnification", use some phrase such as "Magnified region"

> Done.

5) Line 135: Are the authors able to offer any information on differences in the physical location of FSIs connected to TCs vs MCs?

As now reported in our revised manuscript (lines 148-149), and shown in new supplemental figure panels (S11 Fig), MCs and TCs interacted with FSIs spanning comparable positions throughout the EPL.

6) Similarly, the authors show that the connection probability and strength did not differ between FSIs and MCs vs. tufted cells, did they observe any fast lateral inhibition between the two populations?

This is a point of great interest; unfortunately, our experiments were not designed to evaluate this. As now specified in the revised Results: “As slow lateral inhibition is believed to be mediated by at least partially independent TC- and MC-innervating GC subpopulations [Geramita et al., 2016] and has not been reported within mixed TC–MC pairs, we specifically targeted TC pairs and MC pairs, and not TC–MC pairs, for comparison of fast vs. slow lateral inhibition prevalence” (lines 379-382).

In the revised Discussion, we further raise this possibility as motivation for future experiments: “Of further interest, GCs subdivide into superficial- and deep-branching subpopulations selectively innervating TCs and MCs, respectively, to support distinct modes of slow lateral inhibition and contrast enhancement within the two projection neuron populations [Geramita et al., 2016]. While we did not directly test whether individual FSIs can innervate both TCs and MCs, the lack of difference in EPL position between FSIs of synaptically-coupled TC–FSI and MC–FSI pairs, together with the equally high TC and MC connectivity rates observed across the FSI population, intriguingly suggests that FSIs may not only account for the synchronous spontaneous inhibition observed within nearby TC–MC pairs [Arnson and Strowbridge, 2017], but may also mediate fast lateral inhibition between TCs and MCs. Such a link, while speculative, could potentially coordinate fast timescale spike-time patterning across the parallel streams of OB output, and strongly motivates future TC–FSI–MC triplet recordings.” (lines 670-679)

7) There are many instances of "Error! Reference source not found" - please fix

Fixed.

8) In the Discussion section, I wonder if the authors could discuss or speculate on what makes the fast (FSI mediated) and slow lateral inhibition to be mutually exclusive. Were there any specific features (~ distance, location, connected to same glomerulus or different glomeruli) that determine the type of lateral inhibition between MTC pairs?

In the revised Results, we now clarify that while fast and slow lateral inhibition appeared to occur independently, they were not mutually exclusive (lines 400-401), consistent with detection of both fast and slow lateral inhibition in one MTC pair (line 407). However, as suggested by the reviewer, we have also examined intersomatic distances and glomerular separation across MTC pairs (as well as across pairs within each MTC quartet), and interestingly uncovered a particularly high prevalence of fast lateral inhibition specifically between MTCs with directly neighboring somata. This analysis is included in a new supplementary figure (S15 Fig) and is described in the revised Results:

“Post hoc morphological inspection of the above MTC pairs revealed that both fast and slow lateral inhibition occurred independent of whether MTCs connected to directly neighboring glomeruli or glomeruli separated by 1 or ≥2 other glomeruli (S15 Fig). Moreover, intersomatic distance did not significantly differ between MTC pairs exhibiting fast vs. slow lateral inhibition. Examination of each mode of inhibition in isolation, however, revealed that fast lateral inhibition was preferentially detected between nearby MTCs, while no difference in intersomatic distance was found between pairs with vs. without slow lateral inhibition (S15 Fig). This difference arose largely due to a high prevalence of fast, but not slow, lateral inhibition specifically between directly neighboring MTCs (c.f. Fig 4A,C,I; Fig 5A). Indeed, the median intersomatic distance between MTCs exhibiting fast lateral inhibition was zero (S15 Fig). Inspection of an additional cohort of MTC pairs tested for fast lateral inhibition (see below) directly reinforced this pattern, with the probability of detecting fast, but not slow, lateral inhibition exhibiting a pronounced peak (45% of pairs) at zero intersomatic distance (S15 Fig). These results thus further reinforce the conclusion that distinct EPL circuits support fast vs. slow lateral inhibition between heterotypic MTCs, with the high probability for fast lateral inhibition to link directly neighboring MTCs (in addition to other nearby MTCs at lower probability) in good agreement with the spatial dimensions of perisomatic MTC innervation by compact FSI dendritic arbors.” (lines 417-433)
